# USING AN INTERGENERATIONAL RELATIONSHIP TEACHING STRATEGY AND REFLECTION IN ON-GROUND AND ONLINE COURSES

**DOI:** 10.1093/geroni/igz038.3069

**Published:** 2019-11-08

**Authors:** Christine B Thurlow

**Affiliations:** Research College of Nursing, Kansas City, Missouri, United States

## Abstract

Contact or experiences with older adults by college-level students is varied in nature and duration but is often reported as minimal and negative. By pairing groups from different generations, these relationships may influence established attitudes, beliefs, and behaviors of the groups. Research has shown that intergenerational relationships between older adults and college students through interviews within gerontology courses may influence college students’ views on aging and older adults towards the more positive. Specific steps in the development of an intergenerational relationship teaching strategy begin with locating an elder to interview and guidance for how relationship development can change from beginning to end. Role play may help prepare for first interview. Interview questions correspond to course content. Reflections are represented via discussion board if online, and group process forums if on-ground. Intergenerational relationship strategies can be flexible in the amount of contact time with the elder and the student. Reflection strategies can be utilized in several ways: discussion board, journal entries, presentations to classmates, and a culminating paper/PowerPoint. The opportunity for students to tell stories of their own past experiences, relationships with their elders, care and concerns for their elders, and how personal views about older adults and aging may be changing supports the effectiveness of this teaching strategy. Further research into the amount of time needed for effective intergenerational relationship development between student and elder may assist in course planning. Need clearer understanding of the benefits for the elder mentors and if their views about young people have been altered.

